# Case Report: A Metabolic Disorder Presenting as Pediatric Manganism

**DOI:** 10.1289/ehp.10421

**Published:** 2007-08-23

**Authors:** Vanita Sahni, Yves Léger, Linda Panaro, Mark Allen, Scott Giffin, Diane Fury, Nadine Hamm

**Affiliations:** 1 Canadian Field Epidemiology Program, Ottawa, Ontario, Canada; 2 University of Toronto, Community Medicine Program, Toronto, Ontario, Canada; 3 New Brunswick Department of Health, Fredricton, New Brunswick, Canada; 4 New Brunswick Health Region 2, St John, New Brunswick, Canada; 5 New Brunswick Health Region 1, Moncton, New Brunswick, Canada

**Keywords:** environmental medicine, manganese, manganese poisoning, toxicity, water

## Abstract

**Context:**

Manganese is a trace element, essential for physiologic functioning but neurotoxic at high doses. Common exposure sources include dietary intake as well as drinking water in some regions; toxicity is most often associated with inhalation exposures in occupational settings. In this article we describe the investigation of a pediatric case of manganism using both clinical and environmental assessment methods.

**Case presentation:**

A previously healthy 6-year-old child presented with severe Mn neurotoxicity, iron deficiency, and elevated cobalt levels. Immediate and selected extended family members had elevated plasma Mn but remained asymptomatic. An exposure assessment identified seasonal ingestion exposures to Mn at the family’s summer cottage; these were common to the four immediate family members. Well water used for drinking and cooking exceeded recommended guidelines, and foods high in Mn predominated in their diet. No inhalation exposures were identified. Only pica was unique to the patient.

**Discussion:**

The combined evidence of the environmental assessment and biomonitoring of blood Mn levels supported a seasonal ingestion exposure source; this alone was insufficient to explain the toxicity because the patient’s 7-year-old sibling was asymptomatic with almost identical exposures (except pica). A metabolic disorder involving divalent metals (Mn, Fe, and Co) interacting with environmental exposures is the most likely explanation.

**Relevance to clinical or professional practice:**

This case report adds to the emerging body of evidence linking neurologic effects to ingestion Mn exposure.

Manganese is ubiquitous in the environment. It is essential for physiologic functioning in trace quantities, usually supplied by dietary intake. Adverse health effects are typically associated with inhalation exposures within occupational settings, such as the steel or mining industry, and are characterized by neurologic effects [Agency for Toxic Substances and Disease Registry (ATSDR) 2000]. Classical manganism presents with generally irreversible Parkinson-like symptoms (Jiang et al. 2006), which often progress even after exposure ends (Huang et al. 1993). Little is known about the potential health effects resulting from excessive ingestion exposures.

In this report we describe the investigation of a case of pediatric manganism with ingestion exposures. The objectives of this investigation were to confirm the diagnosis, identify the underlying etiology, recommend control measures, and assess whether other persons were at risk.

## Case Presentation

In August 2004, a previously healthy 6-year-old female presented with pica and emotional lability. Over the following months she developed progressive behavioral and neurologic symptoms: She became withdrawn and less verbal with repetitive stuttered speech, and her balance, co-ordination, and fine motor skills declined. By November, she could no longer stand independently, tended to fall backward, and developed a high steppage “cock-like” gait.

Magnetic resonance imaging (MRI) indicated hyperintensity in the basal ganglia, suggesting Mn accumulation. Blood samples analyzed with high-resolution inductively coupled plasma mass spectrometry confirmed elevated levels of Mn in whole blood (39.7 μg/L; normal, 4.3–15.9 μg/L) and serum specimens (3.2 μg/L; normal, 0.3–1.0 μg/L). Severe Fe deficiency [ferritin, 5 μg/L (normal, 12–140 μg/L); serum Fe, 5 μmol/L (normal 5–28 μmol/L)] and polycythemia [red blood cells (RBCs), 7.6 × 10^12^/L; normal, 3.9–5.3 × 10^12^/L) were identified; polycythemia was attributed to elevated Co (3.9 μg/L; normal: 0.7–0.8 μg/L). Blood lead was normal (2.9 μg/dL; normal, 0–4.6 μg/dL) in October 2004 but elevated (17.6 μg/dL) in early August 2005. Investigations failed to determine a cause for the manganism. The patient’s karyotype was normal. A liver biopsy ruled out hepatic dysfunction; however, liver Mn was elevated (34 μg/g; normal, 0.22–4.6 μg/g). Normal blood copper levels ruled out Wilson’s disease.

Treatment included phlebotomies for the polycythemia (November 2004–February 2005, October–November 2005), ethylene-diamine tetraacetic acid chelation for the Mn overload (March–December 2005), and Fe therapy (November 2004–July 2005, October–November 2005). Significant improvements in gait, retropulsion, and motor skills that were observed after the initiation of the phlebotomies and chelation plateaued after a few treatments.

**Figure 1:**
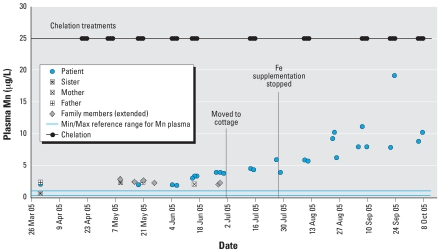
Timeline of Mn levels in plasma for the patient and her family (immediate and extended), March–October 2005. Abbreviations: Max, maximum; Min, minimum.

**Figure 2:**
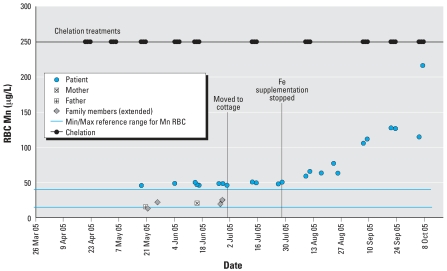
Timeline of Mn levels in RBCs for the patient and her family (immediate and extended), March–October 2005. Abbreviations: Max, maximum; Min, minimum.

In July 2005, Fe supplementation was stopped; a precipitous drop in the patient’s Fe levels immediately followed. Her plasma and RBC Mn levels began rising again (Figures 1 and 2). By August her condition had deteriorated: Her pica returned, she fell frequently, and she needed assistance where she was previously independent. Neurologists estimated that one-fourth of her improvements were lost. Phlebotomies and oral Fe therapy were re-initiated in October.

### Exposure assessment

The patient resided with her mother, father, and 7-year-old sister in an urban center in New Brunswick (NB), Canada. Since 2000, they have spent summers at their nearby cottage—weekend visits in June, followed by full time residence in July and August.

Water testing was conducted; municipal water consumed at the primary residence had nondetectable Mn. At the cottage, a sandpoint well used between 2000 and 2003 had Mn concentrations of 1.7–2.4 mg/L (the health guideline is 0.5 mg/L) (World Health Organization 2006). Spring water and a neighboring cottage well used in 2004 had non-detectable concentrations and 1.7–2.2 mg/L Mn, respectively. In 2005 municipal water was brought to the cottage for drinking, but use of well water for washing and cooking continued.

A food history revealed that the patient and her family consumed more vegetables than a typical Canadian family, particularly Mn-rich leafy green vegetables and pineapples, but they were not vegetarian. Foods consumed by the patient and her sibling were very similar with one exception: The sibling consumed soy milk due to lactose intolerance, whereas the patient consumed dairy. Both took half of a children’s vitamin supplement daily.

No inhalation exposures were identified. No industrial Mn releases were reported in the vicinity of either the primary residence or the cottage (Environment Canada 2003). Neither residence was located in high traffic environments; excessive exposure to methyl-cyclopentadienyl manganese tricarbonyl was therefore unlikely. Parental occupations did not involve exposure to Mn.

Site visits to the primary residence and cottage did not identify excessive exposure sources unique to the patient. No recent renovations, painting, or household products containing Mn were identified. Household members did not participate in hobbies associated with Mn exposure such as glasswork or ceramics. The father infrequently used a welding torch in the detached garage of the primary residence; the children were restricted from the garage during welding but otherwise had free access. During the summer months, vegetables consumed by the patient and her family came predominantly from their cottage garden. Agricultural products containing Mn (e.g., fungicides) were not used. Satellite imagery and site visits did not identify other Mn sources in the vicinity of either residence.

Exposures were very similar for the whole family and virtually identical for the patient and her sister, as they spent the majority of their time together. Only pica was unique to the patient.

### Assessment of other persons at risk

The patient’s parents and sibling were asymptomatic. Between March and June 2005, the immediate family and five extended family members (maternal grandparents, paternal grandfather, and the father’s two siblings in lieu of the deceased paternal grandmother), were tested for Mn. Results indicated elevated plasma Mn ranging from 1.9 to 2.8 μg/L (normal, 0.3–1.0 μg/L) for everyone except the patient’s sister; her plasma Mn was normal (0.6 μg/L) in March but elevated (2.3 μg/L) when retested in May. All those tested had RBC Mn within the normal range (Figures 1 and 2).

Genetic counseling did not identify a familial cause. Both maternal and paternal families are NB Acadian for at least three generations, with no identified consanguinity. No history of Fe deficiency, neurologic disorders (e.g., Parkinson disease), or metabolic disorders were identified.

Early stages of Mn toxicity include muscle tremors and can sometimes resemble Parkinson disease, which has been proposed as a sentinel among selected occupational groups (Harvard Medical School 2000). We examined hospital separation data for Parkinsonism between 1991 and 1995 in which NB rates were similar to other Canadian provinces (Kontakos and Stokes 2000).

Specialists considered this case unique. They queried an international metabolic list-serv to identify other clinically similar cases, and identified a girl with hypermanganesemia of unknown origin in a consanguineous family in the United Kingdom. Her MRI indicated increased intensity in the basal ganglia, and she had a “cock-like” gait. She was polycythemic with elevated iron-binding capacity but had normal serum Fe. We also identified a single case from the literature—a boy from California with polycythemia and manganism explained by liver dysfunction (Gospe et al. 2000).

## Discussion

The patient’s symptoms, biomonitoring results, and environmental assessment were consistent with a chronic, seasonal exposure pattern. Symptom onset occurred in August 2004 with a relapse 1 year later, which was preceded by an increase in blood plasma Mn in early June 2005. The timing of the increase was consistent with findings of increased ingestion exposures at the cottage during the summer months. Unfortunately, continuous biomonitoring of the immediate family’s blood Mn concentrations during the summer was not conducted. A synchronous increase in the family’s Mn levels would have supported the hypothesis that the seasonal cottage exposure was responsible for the patient’s increase in Mn.

The clinical, laboratory, and imaging results support a diagnosis of manganism; however, the patient’s increased Co levels have yet to be explained. Her severe Fe deficiency, unusual for children of her age, is possibly related to these two conditions.

**Figure 3:**
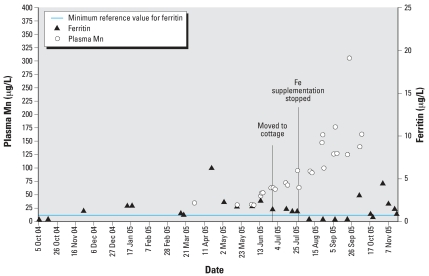
Timeline of ferritin and plasma Mn levels for the patient, October 2004–November 2005.

Co can cause polycythemia through increased erythropoiesis (ATSDR 2004). Phlebotomies used to treat the polycythemia may have exacerbated the Fe deficiency. Low Fe can enhance Mn absorption (Finley 1999; Roth and Garrick 2003); the cessation of Fe supplementation and concurrent depletion of ferritin stores in 2005 possibly contributed to an increased absorption of the Mn at the cottage. However, this does not fully explain the increased blood Mn, as laboratory data clearly indicates that the rise in plasma Mn occurred before the decline in ferritin levels (Figure 3). Ongoing chelation therapy may have also precipitated a release of Mn body stores, contributing to increased blood Mn in the summer and fall of 2005; it remains unclear if this alone could explain the magnitude of the increase.

Females absorb Co and Mn more effectively, and animal studies indicate that the young may absorb more than adults (ATSDR 2000, 2004; Nieboer 2001). Mn, Fe, and Co are divalent cations; all three metals are thought to utilize the same intestinal transporters, and all are essential for proper bodily functioning (ATSDR 2004; Nieboer 2001). An up-regulation of this intestinal transporter is a possible underlying cause of this disorder.

We propose that the presence of a metabolic disorder involving Fe, Mn, and Co, interacting with the cottage environment, may explain the patient’s condition. This may be caused by an underlying genetic or epigenetic defect. Precedents for genetic disorders that are diagnosed after an apparently healthy childhood are numerous (e.g., Tay-Sachs disease, muscular dystrophy). An inherited genetic disorder with variable penetrance or copy-number variations might explain the elevated blood Mn in the asymptomatic extended family members. Acadian backgrounds have been associated with a high incidence of genetic disorders (Lacassie and Flórez 2007); although no parental consanguinity was identified within three generations, more distant family ties cannot be ruled out.

### Other persons at risk

The patient and her immediate family shared very similar high Mn ingestion exposures except for pica; if these exposures alone were responsible for the patient’s adverse health outcome, then her family members would also be expected to exhibit symptoms of Mn toxicity.

Although the immediate and extended family members were asymptomatic, all had elevated plasma Mn (indicative of short-term exposure), with RBC Mn within the normal range (Figures 1 and 2). Elevated plasma Mn may indicate chronic and/or excessive Mn exposure because it is believed that an undetermined exposure threshold must be reached before Mn accumulation occurs in RBC (F. Leung, personal communication). It is unknown whether these markers would consistently precede clinical symptoms and therefore could be used to monitor the patient’s sibling for similar metabolic deterioration.

Well water in NB and other Maritime provinces commonly exceeds the health-based drinking water guidelines for Mn due to the underlying geology (New Brunswick Department of Environment and Local Government 2004). Because generations of rural residents have relied on groundwater containing high Mn concentrations, it seems that selective pressures would have previously highlighted any genetic disorders that disrupt normal Mn homeostasis. Yet pediatric specialists considered this case presentation unique. Also, provincial Parkinsonism rates were comparable to the rest of Canada (Kontakos and Stokes 2000); this crude indicator suggests early manganism symptoms were not artificially inflating the Parkinson disease rates in NB.

The literature suggests a spectrum of disease, from the more severe forms of manganism to more subtle neurologic health effects. Some studies have reported associations between Mn exposure in drinking water and reported cognitive/behavioral problems in children in Quebec (Bouchard et al. 2007), Bangladesh (Wasserman et al. 2006), and Tawain (He et al. 1994). Similar long-term Mn exposure studies in adults have been mixed, with some researchers describing neurologic dysfunction with increasing levels of Mn in drinking water (Kondakis et al. 1989) while others found no neurologic differences (Vieregge et al. 1995). The true health burden of excessive Mn ingestion is currently unknown.

### Limitations

There currently is no “gold standard” biomarker for Mn exposure. The primary care team used plasma and RBC Mn as indicators. These are effective tools in measuring exposure differences between populations, but they are less reliable on an individual level (Apostoli et al. 2000; Bader et al. 1999; Institute for Environment and Health 2004; Mergler 1999; Ostiguy et al. 2003). Whole blood may be the best indicator for an individual (Takagi et al. 2002). It remains unclear to what extent observed variations in a biomarker represent real variations in exposure. For example, three consecutive blood samples for plasma Mn taken from the patient at the hospital on a single day showed large variability: 0.6, 2.2, and 2.4 μg/L. Comparing measurements between individuals may be subject to the same variability.

A lack of appropriate population reference values further complicated the interpretation of Mn blood tests. Reference ranges were derived from population norms in southern Ontario; the absence of available reference levels for plasma and RBC Mn for populations exposed to higher natural background levels (e.g., through drinking water) makes the interpretation of such results difficult. The seemingly elevated plasma Mn levels among the patient’s family members may well be within a “normal” range.

There is also no “gold standard” for measuring health effects from excessive Mn. Nonspecific neurobehavioral symptoms may be overlooked or misdiagnosed. Members of the patient’s immediate and extended family had no overt symptoms attributable to manganism, but they were not tested for oppositional behavior and hyperactivity (Bouchard et al. 2007), IQ (Wasserman et al. 2006), short-term verbal and visual memory (Woolf et al. 2002), or manual dexterity and visuo-perceptive speed (He et al. 1994). Which tests are best at detecting which health effects in which populations is a nascent field of investigation.

## Conclusion and Recommendations

The available evidence suggests that environmental exposures alone are unlikely to be the sole cause of this child’s illness; however, an unidentified exposure as a result of her pica cannot be excluded. A pervasive metabolic disorder (Mn, Fe, Co) is a more plausible explanation. This hypothesis could incorporate both the environmental exposures and clinical/laboratory findings, but is limited because the proposed underlying genetic defect cannot be identified at this time.

Given the patient’s deteriorating clinical status, we recommended that the family use only municipal water because boiling foods in the Mn-rich well water may concentrate this cation (British Columbia Ministry of the Environment 2007; Bouchard et al. 2007). Also, the garage used for welding should always be off-limits to the children. Finally, controlling the pica and implementing a low-Mn, Fe-rich diet in addition to ongoing Fe supplementation would be helpful.

In addition to these patient-specific control measures, the broader issue of blood Mn reference levels remains to be addressed. The Canadian Health Measures Survey, which will collect blood and urine samples from 5,000 randomly chosen adults from 15 cities across Canada, begins its data collection in the winter of 2007 (Statistics Canada 2006). Mn is among the metals to be measured in the survey participants.

## References

[b1-ehp0115-001776] Apostoli P, Lucchini R, Alessio L (2000). Are current biomarkers suitable for the assessment of manganese exposure in individual workers?. Am J Ind Med.

[b2-ehp0115-001776] ATSDR (2000). Toxicological Profile for Manganese. Atlanta, GA: Agency for Toxic Substances and Disease Registry.

[b3-ehp0115-001776] ATSDR (2004). Toxicological Profile for Cobalt. Atlanta, GA: Agency for Toxic Substances and Disease Registry.

[b4-ehp0115-001776] Bader M, Dietz M, Ihrig A, Triebig G (1999). Biomonitoring of manganese in blood, urine and axillary hair following low-dose exposure during the manufacture of dry cell batteries. Int Arch Occup Environ Health.

[b5-ehp0115-001776] Bouchard M, Laforest F, Vandelac L, Bellinger D, Mergler D (2007). Hair manganese and hyperactive behaviors: pilot study of school-age children exposed through tap water. Environ Health Perspect.

[b6-ehp0115-001776] British Columbia Ministry of the Environment (2007). Iron and Manganese in Groundwater. Water Stewardship Information Series.

[b7-ehp0115-001776] Environment Canada (2003). National Pollutant Release Inventory.

[b8-ehp0115-001776] Finley JW (1999). Manganese absorption and retention by young women is associated with serum ferritin concentration. Am J Clin Nutr.

[b9-ehp0115-001776] Gospe SM, Caruso RD, Clegg MS, Keen CL, Pimstone NR, Ducore JM (2000). Paraparesis, hypermanganesaemia, and polycythaemia: a novel presentation of cirrhosis. Arch Dis Child.

[b10-ehp0115-001776] Harvard Medical School (2000). Sentinel health effects, occupational diagnosis with known environmental exposure as etiological agents. Occupational and Environmental Medicine.

[b11-ehp0115-001776] He P, Liu DH, Zhang GQ (1994). Effects of high-level-manganese sewage irrigation on children’s neurobehaviour [in Chinese]. Zhonghua Yu FangYi Xue Za Zhi.

[b12-ehp0115-001776] Huang CC, Lu CS, Chu NS, Hochberg F, Lilienfield D, Olanow W (1993). Progression after chronic manganese exposure. Neurology.

[b13-ehp0115-001776] Institute for Environment and Health (2004). Occupational Exposure Limits: Criteria Document for Manganese and Inorganic Manganese Compounds. IEH Web Report W17.

[b14-ehp0115-001776] Jiang YM, Mo XA, Du FQ, Fu X, Zhu XY, Gao HY (2006). Effective treatment of manganese-induced occupational Parkinsonism with *p*-aminosalicylic acid: a case of 17-year follow up study. J Occup Environ Med.

[b15-ehp0115-001776] Kondakis X, Makris N, Leotsinidis M, Prinou M, Papapetropoulos T (1989). Possible health effects of high manganese concentration in drinking water. Arch Environ Health.

[b16-ehp0115-001776] Kontakos N, Stokes J (2000). Monograph Series on Aging-related Diseases: XII. Parkinson’s Disease—Recent Developments and New Directions. Chronic Diseases in Canada 20(3).

[b17-ehp0115-001776] Lacassie Y, Flórez L (2007). Some Genetic Disorders among Acadian People.

[b18-ehp0115-001776] Mergler D (1999). Neurotoxic effects of low level exposure to manganese in human populations. Environ Res.

[b19-ehp0115-001776] New Brunswick Department of Environment and Local Government (2004). Manganese: New Brunswick Well Water Quality Distribution Atlas.

[b20-ehp0115-001776] Nieboer E (2001). Toxicological Profile and Related Health Issues: Cobalt (for Physicians).

[b21-ehp0115-001776] Ostiguy C, Malo S, Asselin P (2003). Synthesis of Scientific Knowledge on the Health Risks following Occupational Exposure to Manganese.

[b22-ehp0115-001776] Roth J, Garrick M (2003). Iron interaction and other biological reactions mediating the physiological and toxic actions of manganese. Biochem Pharmacol.

[b23-ehp0115-001776] Statistics Canada (2006). Canadian Health Measures Survey.

[b24-ehp0115-001776] Takagi Y, Okada A, Sando K, Wasa M, Yoshida H, Hirabuki N (2002). Evaluation of indexes of in vivo manganese status and the optimal intravenous dose for adult patients undergoing home parenteral nutrition. Am J Clin Nut.

[b25-ehp0115-001776] Vieregge P, Heinzow B, Korf G, Teichert HM, Schleifenbaum P, Mösinger H (1995). Long term exposure to manganese in rural well water has no neurological effects. Can J Neurol Sci.

[b26-ehp0115-001776] Wasserman GA, Liu X, Parvez F, Ahsan H, Levy D, Factor-Litvak P (2006). Water manganese exposure and children’s intellectual function in Araihazar, Bangladesh. Environ Health Perspect.

[b27-ehp0115-001776] Woolf A, Wright R, Amarasiriwardena C, Bellinger D (2002). A child with chronic manganese exposure from drinking water. Environ Health Perspect.

[b28-ehp0115-001776] World Health Organization (2006). Guidelines for Drinking Water Quality. First Addendum to Third Edition. Volume 1: Recommendations. Geneva:World Health Organization.

